# How perceived parental educational aspirations predict primary school children's educational aspirations, mathematics achievement and social-emotional competence: the mediating role of mathematics self-beliefs

**DOI:** 10.3389/fpsyg.2025.1633737

**Published:** 2025-09-23

**Authors:** Jiaqi Yang, Yehui Wang

**Affiliations:** Collaborative Innovation Center of Assessment for Basic Education Quality, Beijing Normal University, Beijing, China

**Keywords:** perceived parental educational aspirations, educational aspirations, mathematics achievement, social-emotional competence, mathematics self-beliefs

## Abstract

**Introduction:**

Children's perceived parental educational aspirations play an important role in their development. However, few studies have examined the relationships between children's perceived parental educational aspirations and their own educational aspirations, mathematics achievement, and social-emotional competence (SEC), as well as the mediating role of mathematics self-beliefs in these relationships. This study investigated that how children's perceived parental educational aspirations were transmitted into their subsequent educational aspirations, mathematics achievement, and SEC through mathematics self-beliefs.

**Method:**

Participants were 3,995 fourth-grade students in China (47.06% girls; *M age* = 10.76 years, *SD* = 0.90) selected through cluster random sampling method. Of these, 2,789 were followed up two years later. Students completed a questionnaire that included background information and scales measuring mathematics self-beliefs and SEC, as well as a mathematics achievement test. Structural equation modeling was conducted to examine the predictive effects of children's perceived parental educational aspirations on their educational aspirations, mathematics achievement, and SEC, and the mediating role of mathematics self-beliefs.

**Results:**

Results indicated that perceived parental educational aspirations positively predicted mathematics self-beliefs (β = 0.13, *p* < 0.001), educational aspirations (β = 0.25, *p* < 0.001), mathematics achievement (β = 0.26, *p* < 0.001), and SEC (β = 0.14, *p* < 0.001). Mathematics self-beliefs also positively predicted educational aspirations (β = 0.29, *p* < 0.001), mathematics achievement (β = 0.45, *p* < 0.001), and SEC (β = 0.45, *p* < 0.001).

**Discussion:**

These findings highlight the benefits of children's perceived parental educational aspirations for their development and the central role of mathematics self-beliefs. They suggest that when parents demonstrate positive educational expectations, children are more likely to develop positive self-beliefs, which in turn foster higher aspirations, stronger academic performance, and enhanced SEC.

## 1 Introduction

Parental educational aspirations refer to the level of education that parents hope their children will attain. Such beliefs have been found to be closely associated with children's academic achievement (Šimunović and Babarović, 2020), educational aspirations (Schörner and Bittmann, 2023), and social-emotional development (Wang et al., 2022). Students' educational aspirations and achievement are positively related to a range of academic outcomes, including learning motivation, school performance, and occupational attainment (Adler et al., 2024; Khattab et al., 2022). Moreover, social-emotional competence (SEC) has increasingly been recognized as a core component of school education alongside academic development in contemporary pedagogical frameworks. SEC encompasses a broad range of abilities, including managing goal- and task-oriented behaviors, establishing and maintaining positive social relationships, and regulating emotions, playing a critical role in children's overall development (Soto et al., 2022; Tang et al., 2023).

According to the two-step model of value transmission, this process unfolds in two stages (Grusec and Goodnow, 1994). First, children must accurately perceive parental values; second, they must accept these values as their own. Similarly, expectancy-value theory (EVT) posits that children's expectancy of success and value beliefs jointly predict achievement-related choices and performance. These beliefs are shaped, in part, by parents' and teachers' attitudes and behaviors (Eccles and Wigfield, 2002; Fredricks and Eccles, 2002). The two theories suggest that accurate perception of parental values is a necessary precondition for internalization. Without conscious awareness and cognitive representation of parental values, children lack the perspective required for acceptance and consequently, maybe unable to develop positive self-beliefs. Taken together, the theories highlight three pathways: (a) children perceive parental beliefs, (b) perceived parental beliefs shape children's self-beliefs, and (c) these internalized self-beliefs ultimately predict achievement-related outcomes.

Children's perceived parental educational aspirations reflect the level of education that children believe their parents want them to attain. Recent empirical studies have demonstrated associations between parental education aspirations and children's perceptions of these aspirations, as well as between perceived parental aspirations and children's own aspirations (Chen et al., 2022; Schörner and Bittmann, 2023). The present study aimed to extend this line of research by examining the associations between children's perceived parental educational aspirations and their broader development. Specifically, we investigated how perceived parental educational aspirations influence children's subsequent educational aspirations, mathematics achievement, and SEC two years later, through the mediating role of self-beliefs. Exploring these longitudinal relationships provides valuable insights into the educational and developmental trajectories of primary school students.

### 1.1 Benefits of perceived parental educational aspirations

#### 1.1.1 The relationship between perceived parental educational aspirations and children's educational aspirations

The Wisconsin Social Psychological Model of Status Attainment (WIM; Sewell and Hauser, 1980; Sewell et al., 2003) posits that, in addition to structural factors (e.g., family background, years of education), socio-psychological factors—particularly “significant others” (such as parents and teachers) and “self-aspirations”—play crucial roles in educational investment and academic achievement. Parents are pivotal in shaping children's values, self-perceptions and aspirations (Eccles and Wigfield, 2002). Empirical studies have shown that parental educational aspirations are positively associated with children's educational aspirations (Schörner and Bittmann, 2023). Moreover, the two-step model of value transmission (Grusec and Goodnow, 1994) underscores the importance of children's accurate perception of parental beliefs. Both theoretical and empirical perspectives support the view that children's perceived parental aspirations contribute significantly to the development of their own educational aspirations.

#### 1.1.2 The relationship between perceived parental educational aspirations and children's achievement

Several studies have highlighted the importance of children's perceptions of their parents' attitudes and behaviors. For example, children's perceived parental involvement has been found to be significantly associated with their academic achievement (Cui et al., 2024; Eker and Yildizli, 2025). However, further research is needed to clarify the relationship between children's perceived parental educational aspirations and their academic achievement. Prior studies have shown that parental educational aspirations serve as a motivational force that promotes children's subsequent achievements in mathematics (e.g., Šimunović and Babarović, 2020). Moreover, evidence suggests a reciprocal relationship between children's perceived parental educational aspirations and parents' aspirations for their children (Schörner and Bittmann, 2023). Based on previous studies (e.g., Schörner and Bittmann, 2023; Šimunović and Babarović, 2020), we hypothesize that children's perceived parental educational aspirations positively predict their subsequent mathematics achievement.

#### 1.1.3 The relationship between perceived parental educational aspirations and children's SEC

SEC contributes to individuals' development across various domains, including educational and social outcomes, as well as mental and physical health (Harris et al., 2022; Tang et al., 2023). Parental beliefs (e.g., valuing play-based interactions or diverse educational activities) and parental expectations have been shown to play a significant role in children's social-emotional development (Li et al., 2023; Liu et al., 2021; Wang et al., 2022). Drawing on the WIM (Sewell and Hauser, 1980; Sewell et al., 2003) and the two-step model of value transmission (Grusec and Goodnow, 1994), it can be assumed that children's perceived parental values may predict their subsequent SEC. However, little is known about whether children's perceived parental educational aspirations are associated with their SEC. Investigating this relationship may yield new insights into the broader developmental benefits of children's perceived parental educational aspirations.

### 1.2 The mediating role of self-beliefs

The EVT emphasizes the importance on children's self-beliefs (Eccles and Wigfield, 2002; Fredricks and Eccles, 2002), which play a significant role in promoting academic achievement (Watson et al., 2024). Self-beliefs are a multidimensional construct (Bandura, 1997), encompassing self-efficacy, self-concept, self-confidence, and anxiety (Sakellariou, 2020; Stankov and Lee, 2017; Zhu and Meyer, 2022). Self-efficacy reflects an individual's belief in his or her ability to successfully perform specific tasks, whereas self-concept represents a broader perception of one's personal attributes based on continuous self-evaluation (OECD, 2013; Pedrero and Manzi, 2020). Self-confidence refers to the belief in one's abilities, strengths, and judgment, while anxiety captures the physiological and emotional responses associated with testing or with a task-related situations (Smith et al., 2025). Collectively, these dimensions of self-beliefs have been identified as reliable predictors of academic success and future life outcomes (Stankov and Lee, 2017).

#### 1.2.1 The relationship between perceived parental educational aspirations and children's self-beliefs

Children's self-perceptions are closely linked to their parents' attitudes and beliefs (Eccles and Wigfield, 2002). Among various parental beliefs, educational aspirations show a stable and robust association with children's development (Šimunović and Babarović, 2020). Parents communicate these aspirations directly by encouraging children to perform well at school or providing positive feedback for their achievement (Hoferichter et al., 2021). They also convey their beliefs indirectly through time spent with children, such as assisting with schoolwork (Falanga et al., 2023). Through these direct and indirect processes, children form perceptions of their parents' educational aspirations, which, according to EVT, contribute to the development of their self-beliefs (Eccles and Wigfield, 2002).

#### 1.2.2 The relationship between self-beliefs and educational aspirations, achievement, and SEC

Positive self-beliefs have been found to benefit multiple domains of development, including academic, social and emotional outcomes (Frazee, 2023). For instance, those students with positive self-beliefs tend to have better academical performance (Watson et al., 2024) and are more likely to set ambitious educational goals (Du et al., 2021). Similarly, (Loos-Sant'Ana and Trancoso 2014) found that students with positive beliefs exhibited greater social competence. In contrast, negative self-beliefs may lead to frustration and increased withdrawal behaviors. These findings suggest that self-beliefs may serve as a key predictor of children's educational aspirations, academic achievement, and SEC.

### 1.3 The present study

Although existing studies have demonstrated the benefits of parental educational aspirations for children's academic achievement and educational aspirations (Schörner and Bittmann, 2023; Šimunović and Babarović, 2020), relatively few studies have examined the effects of children's perceived parental educational aspirations (Schörner and Bittmann, 2023). Understanding how children's perceived parental educational aspirations relate to their own educational aspirations, achievement, and SEC is essential for clarifying the role of children's perceptions of parental aspirations. Moreover, self-beliefs may serve as a key mediator between perceived parental educational aspirations and children's own aspirations (Eccles and Wigfield, 2002; Fredricks and Eccles, 2002). This is particularly relevant for primary school children, whose self-beliefs are undergoing rapid development (Jansen et al., 2024). Investigating the mediating role of self-beliefs can provide deeper insights into the pathways through which children's perceived parental educational aspirations predict their subsequent educational aspirations, mathematics achievement, and SEC.

**Figure 1:**
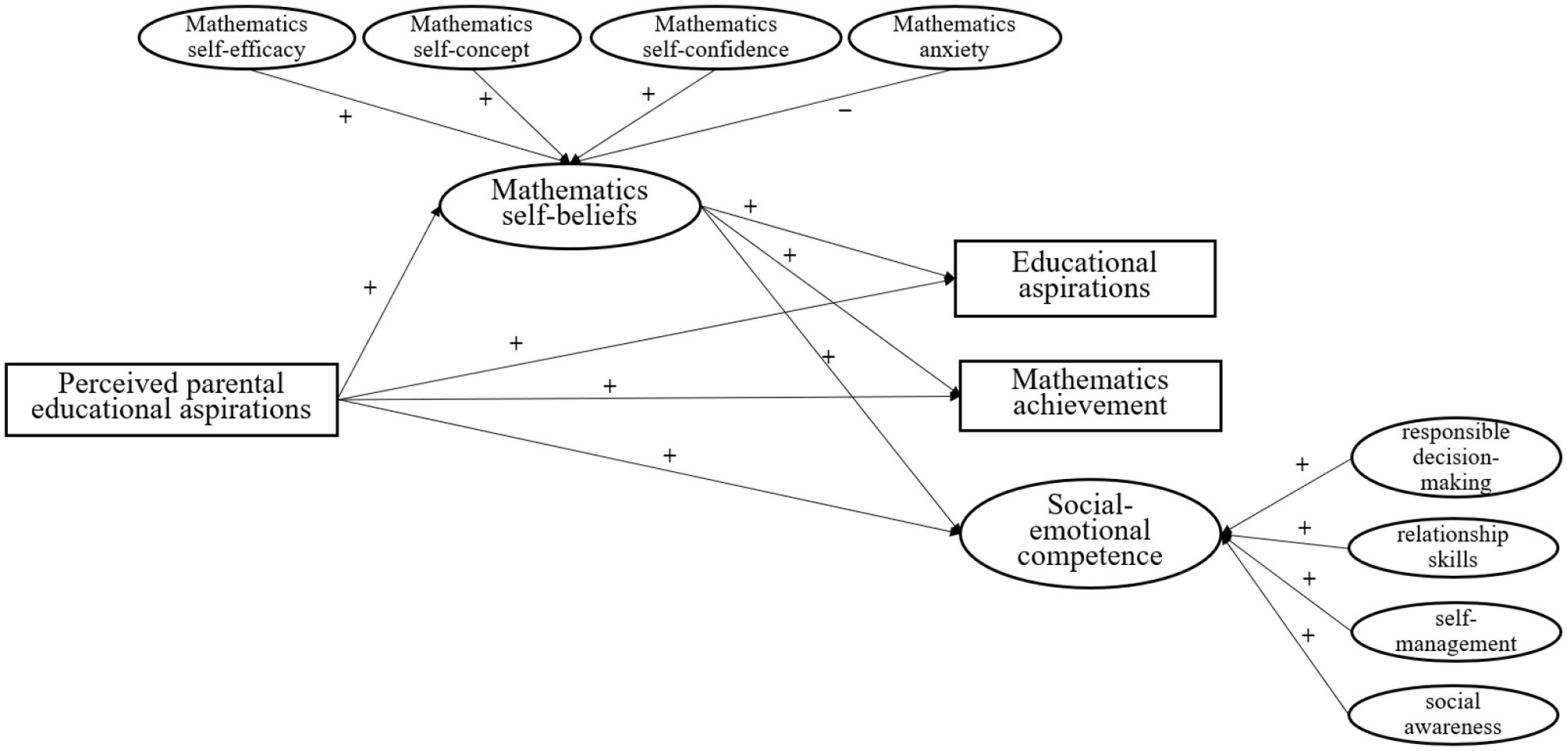
Hypothesized path diagram: the mediation of mathematics self-beliefs on predicting relationships between perceived parental educational aspirations and primary school children's educational aspirations, mathematics achievement and SEC. Mathematics achievement and educational aspirations are observable variables, represented by rectangles in this figure. Mathematics self-beliefs and social-emotional competence are latent variables, which are denoted as ellipses. The symbol “+” represents positive relationships. The symbol “–” represents negative relationships.

The present study focuses on mathematics because it is particularly challenging for primary school students, often eliciting stronger emotional responses (e.g., anxiety) and more pronounced self-belief patterns (Demedts et al., 2022). Moreover, mathematics-related beliefs strongly predict long-term STEM outcomes and are recognized as critical for later development (Kwon et al., 2021). Improving students' attitudes, emotions and beliefs in mathematics is also a stated goal of the *Mathematics Curriculum Standards for Compulsory Education* (2022 edition; Ministry of Education of the People's Republic of China, 2022). The hypothesized framework is presented in Figure 1. Accordingly, the present study seeks to address the following research questions (RQs):

RQ1: Do children's perceived parental educational aspirations directly predict their educational aspirations, mathematics achievement, and SEC?

RQ2: Do children's mathematics self-beliefs directly predict their educational aspirations, mathematics achievement, and SEC?

RQ 3: Do children's mathematics self-beliefs indirectly (i.e., as the mediator) predict the associations between perceived parental educational aspirations and children's educational aspirations, mathematics achievement, and SEC?

## 2 Method

### 2.1 Participants and procedure

Grades 4–6 represent a critical period for mathematics learning, during which students encounter increasingly abstract content in mathematics (e.g., fractions, probability) and heightened level of difficulty. These challenges can significantly shape their cognitive development and self-beliefs (Demedts et al., 2022). In addition, as socialization process intensifies during this stage, students' SEC also undergoes important transformations (Deng et al., 2025; Yang et al., 2023). Early adolescence is therefore a pivotal developmental period with long-term implications for children's future trajectories. To better understand the role of children's perceived parental educational aspirations in their development, this study employed a two-wave longitudinal design. Specifically, it examined how fourth-grade students' perceived parental educational aspirations predicted their mathematics self-beliefs, educational aspirations, mathematics achievement, and SEC two years later, as well as the mediating role of mathematics self-beliefs.

Cluster random sampling was employed, whereby schools were first randomly selected, and then one intact fourth-grade class from each selected school was chosen for assessment. The final sample at Time 1 (T1) consisted of 3,995 Chinese fourth-grade students (47.06% girls; *M age* = 10.76 years, *SD* = 0.90) from 99 schools. On average, each school contributed 1.02% of the total sample (range: 0.3%−1.9%). Two years later, at Time 2 (T2), 2,789 students (46.8% girls, *M age* = 12.43 years, *SD* = 1.18) were successfully followed. Attrition was primarily due to schools merges, student transfers, and illness.

At T1, students were required to report their perceived parental educational aspirations and background information (including gender, left-behind status, parents' highest level of education, home possessions, number of books in the home, and parental investment in education). At T2, students' mathematics self-beliefs (mathematics self-efficacy, mathematics self-concept, mathematics self-confidence and mathematics anxiety), educational aspirations, mathematics achievement, and SEC were assessed. Both assessments were administered in paper-and-pencil format. Completion time was approximately 15 min for the questionnaire and 40 min for the mathematics achievement test. Informed consent was obtained from schools principals, parents, and students. All participants were informed of the voluntary and anonymous nature of the study and their right to withdraw at any time.

### 2.2 Measures

#### 2.2.1 Perceived parental educational aspirations and educational aspirations

Children's perceived parental educational aspirations were assessed using a single item: “*How far in school do you think your parents want you to go?*” Children's own educational aspirations were measured with a parallel item: “*How far in school do you want to go?*” Both items were adapted from the Programme for International Student Assessment (PISA) 2012 (OECD, 2014). The use of single-item measures has been supported in prior research (e.g., OECD, 2014; Schörner and Bittmann, 2023). Responses were recorded on a five-point scale (1 = primary school, 2 = junior high school, 3 = senior high school, 4 = bachelor's degree, 5 = graduate degree).

#### 2.2.2 Mathematics self-beliefs

Children's mathematics self-beliefs were modeled as a latent construct comprising four components—mathematics self-efficacy, mathematics self-concept, mathematics self-confidence, and mathematics anxiety—based on Stankov and Lee's (2017) framework. The scales used to measure these dimensions were adapted from PISA 2012, which was initially developed for 15-year-old students (OECD, 2014). To ensure developmental appropriateness and cultural relevance for primary school students in China, the scales were carefully adapted and translated. A pilot test and cognitive interviews were conducted, confirming that 10-year-old children were able to comprehend the items.

##### 2.2.2.1 Mathematics self-efficacy

Mathematics self-efficacy was measured with an eight-item scale (e.g., “*Estimating the approximate length of 1,000 steps*”), in which students rated how confident they felt about completing each task. Responses were given on a four-point scale (1 = not at all confident, 2 = not very confident, 3 = confident, 4 = very confident). In the present study, the scale demonstrated good internal consistency (Cronbach's α = 0.87). Confirmatory factor analysis (CFA) indicated an acceptable model fit (χ^2^/*df* = 9.81; RMSEA = 0.051; CFI = 0.982; TLI = 0.975; SRMR = 0.021).

##### 2.2.2.2 Mathematics self-concept

Mathematics self-concept was measured with a five-item scale (e.g., “*I am good at solving difficult mathematical problems*”). Students responded on a four-point scale (1 = strongly disagree, 2 = disagree, 3 = agree, 4 = strongly agree). The scale demonstrated good internal consistency (Cronbach's α = 0.84). The results of CFA indicated that the model fitted the data well (χ^2^/*df* = 44.40; RMSEA = 0.081; CFI = 0.970; TLI = 0.941; SRMR = 0.027).

##### 2.2.2.3 Mathematics self-confidence

Mathematics self-confidence was assessed using a four-item scale (e.g., “*I learn mathematics very quickly*”). Responses were rated on a four-point scale (1 = strongly disagree, 2 = disagree, 3 = agree, 4 = strongly agree). In this study, the scale demonstrated good internal consistency (Cronbach's α = 0.90). The results of CFA indicated that the model fitted the data well (χ^2^/*df* = 3.72; RMSEA = 0.028; CFI = 0.999; TLI = 0.998; SRMR = 0.004).

##### 2.2.2.4 Mathematics anxiety

Mathematics anxiety was assessed using a five-item scale (e.g., “*I often worry that mathematics will be difficult*”). Responses were rated on a four-point scale (1 = strongly disagree, 2 = disagree, 3 = agree, 4 = strongly agree). The scale had good internal consistency (Cronbach's α = 0.84). The results of CFA indicated that the model fitted the data well (χ^2^/*df* = 36.97; RMSEA = 0.083; CFI = 0.973; TLI = 0.945; SRMR = 0.029).

##### 2.2.2.5 Second-order mathematics self-beliefs

The second-order latent construct of mathematics self-beliefs demonstrated acceptable composite reliability (CR = 0.76). The CFA results of the model, comprising the four subconstructs, indicated good fit: χ^2^/*df* = 10.72, RMSEA = 0.053, CFI = 0.936, TLI = 0.926, SRMR = 0.059. All second-order factor loadings were substantial (absolute values > 0.35), with 0.68 for mathematics self-efficacy, 0.97 for mathematics self-concept, 0.94 for mathematics self-confidence, and −0.39 for mathematics anxiety.

#### 2.2.3 Mathematics achievement

Children's mathematics achievement was assessed using a test specifically developed for primary school students in grades 4–6. The test was designed collaboratively by teachers and educational measurement experts in accordance with the *Mathematics Curriculum Standards for Primary Education and Junior Secondary Education* (2011). It consisted of 41 multiple-choice items covering the content strands of number and algebra, space and shape, and statistics and probability. Students were given 40 min to complete the test. Scores were estimated using the Rasch model (Bond and Fox, 2013) and converted to a 0–100 scale. The test demonstrated good internal consistency in this study (Cronbach's α = 0.89). The average item difficulty coefficient was 0.20, ranging from −1.66 to 2.27.

#### 2.2.4 Social-emotional competence

Children's SEC was measured using the Chinese version of the Delaware Social and Emotional Competency Scale, initially developed for primary, middle, and high school students (Mantz et al., 2016). The scale has been validated for use with primary school students in China (e.g., Deng et al., 2025; Yang et al., 2023; Zhu et al., 2024). SEC was assessed across four dimensions: responsible decision-making (e.g., “*I blame others when I'm in trouble*”), social awareness (e.g., “*I care about how others feel*”), self-management (e.g., “*I think before I act*”), and relationship skills (e.g., “*I am good at solving conflicts with others*”). Each dimension included 3 items, rated on a five-point scale (1 = not like me at all to 5 = very much like me). The Cronbach's alpha coefficient for the total scale was 0.83, while the values for the four subscales were 0.62, 0.67, 0.62, and 0.57, respectively. According to Mantz et al. (2016), the low alpha coefficients of the four subscales were largely attributed to only three items per subscale. Given these low coefficients, it is not recommended to use subscale scores. Thus, the four dimensions were conceptualized as indicators of a higher-order SEC factor in the analytical model. Moreover, CFA indicated acceptable model fit (χ^2^/*df* = 13.48; RMSEA = 0.060; CFI = 0.935; TLI = 0.914; SRMR = 0.034).

#### 2.2.5 Control variables

In the present study, over 30% of participants were left-behind children. These are children under the age of 18 who remain at their registered household location while one or both parents migrate elsewhere for work, resulting in living apart for at least 6 months (National Bureau of Statistics et al., 2023). This demographic characteristic has been linked to children's academic achievement (Zhang and Huang, 2022) and was therefore included as a control variable. Children's socioeconomic status (SES) was also controlled, given its established association with children's academic achievements and development (Liu et al., 2022; Li et al., 2025). SES was derived from the first principal component via principal component analysis (PCA) of four indicators: parents' highest level of education, home possessions, number of books in the home, and parental investment in education, following OECD guidelines (OECD, 2014). Additionally, children's gender was included as a control variable, as prior research has shown its relevance to academic outcomes (Wang and Yu, 2023).

### 2.3 Statistical analysis

Preliminary analyses, including Harman's single-factor test, descriptive statistics, Pearson correlations, and reliability analysis, were conducted using SPSS 24. Outlier were screened using standardized *Z*-scores. Cases beyond |3.0| standard deviations were flagged, resulting in 61 outliers for children's perceived parental educational aspirations and 17 outliers for children's educational aspirations. Sensitivity analyses excluding these outliers produced results that were substantively consistent with the primary model. The SEM results without outliers are presented in Appendix.

Independent-samples *t* tests were conducted to assess the longitudinal attrition by comparing participants who completed both T1 and T2 (completers) with those who participated only at T1 (non-completers). CFA was then performed to evaluate the fit of the multidimensional structure of mathematics self-beliefs. Finally, structural equation modeling (SEM) was conducted using Mplus 8.3 to address research questions. In the SEM, children's perceived parental educational aspirations at T1, along with control variables (gender, SES, and left-behind status), served as independent variables. Mathematics self-beliefs at T2, comprising four components (mathematics self-efficacy, mathematics self-concept, mathematics confidence, and mathematics anxiety), served as the mediator. Children's educational aspirations, mathematics achievement, and SEC at T2 were modeled as dependent variables.

A robust maximum likelihood estimator (MLR) was used to estimate the SEM results. Although the chi-square test (χ^2^) is commonly used to evaluate model fit, it is sensitive to sample size. Therefore, additional fit indices were considered, including the comparative fit index (CFI), Tucker-Lewis index (TLI), root mean square error of approximation (RMSEA), and standardized root mean square residual (SRMR). Following conventional guidelines, CFI and TLI values above 0.95 indicate good fit, whereas values between 0.90 and 0.95 are considered acceptable. RMSEA values below 0.06 indicate good fit, with values between 0.06 and 0.08 deemed acceptable (Hu and Bentler, 1999). SRMR values below 0.05 are indicative of good model fit (McDonald and Ho, 2002).

## 3 Results

### 3.1 Preliminary results

Harman's single-factor test was conducted on the full dataset to examine the potential common method bias. Results indicated six factors with eigenvalues greater than 1, with the first factor accounting for 28.30% of the total variance —well below the critical threshold of 50% (Podsakoff et al., 2003). These findings suggest that common method bias was not a concern in the present study.

Longitudinal attrition was examined using independent-samples *t* tests. No statistically significant differences were found between completers (participants at both T1 and T2) and non-completers (T1 only) in gender [*t*_(3984)_ = 2.02, *p* > 0.05], left-behind status [*t*_(3243)_ = −0.95, *p* > 0.05], or SES [*t*_(1908)_ = −1.67, *p* > 0.05]. Although a significant difference emerged in children's perceived parental educational aspirations between completers and non-completers [*t*_(3287)_ = 3.53, *p* < 0.05], the effect size was small (d = 0.14). Missing data were handled using the full information maximum likelihood (FIML) method, which produces unbiased and efficient parameter estimates (Newsom, 2015).

Descriptive statistics and Pearson correlations for the main variables are presented in Tables 1, 2, respectively. Standardized skewness and kurtosis values ranged from −3 to +3, indicating that the variables were approximately normally distributed (Brown, 2006). Normality was also examined at the item level, with skewness values ranging from −1.82 to 0.71 and kurtosis values ranging from −2.00 to 2.15, further supporting the appropriateness of the data for parametric analyses.

**Table 1:** Descriptive statistics.

**Variables**	***M***	***SD***	**Min**	**Max**	**Skewness**	**Kurtosis**	***N***
Perceived parental educational aspirations (T1)	4.33	0.84	1.00	5.00	−1.73	3.88	3,142
Mathematics achievement (T2)	51.00	17.80	0.78	95.82	−0.06	−0.49	2,784
Educational aspirations (T2)	4.23	0.82	1.00	5.00	−1.17	1.51	2,552
Mathematics self-efficacy (T2)	3.01	0.65	1.00	4.00	−0.31	−0.19	2,727
Mathematics self-concept (T2)	2.59	0.78	1.00	4.00	−0.02	−0.67	2,740
Mathematics self-confidence (T2)	2.54	0.82	1.00	4.00	−0.01	−0.61	2,759
Mathematics anxiety (T2)	2.29	0.82	1.00	4.00	0.13	−0.72	2,728
SEC—responsible decision-making (T2)	3.04	0.65	1.00	4.00	−0.40	−0.42	2,648
SEC—social awareness (T2)	2.92	0.75	1.00	4.00	−0.50	−0.26	2,638
SEC—self-management (T2)	3.01	0.73	1.00	4.00	−0.61	−0.09	2,640
SEC—relationship skills (T2)	3.04	0.70	1.00	4.00	−0.72	0.28	2,637

T1 represents the time point of initial data collection, and T2 denotes the follow-up survey conducted 2 years later. SEC is social-emotional competence. *M*, mean; *SD*, standard deviation; Min, minimum; Max, maximum.

**Table 2:** Pearson correlations.

**Variables**	**1**	**2**	**3**	**4**	**5**	**6**	**7**	**8**	**9**	**10**
1 Perceived parental educational aspirations (T1)	1									
2 Mathematics achievement (T2)	0.33^***^	1								
3 Educational aspirations (T2)	0.32^***^	0.34^***^	1							
4 Mathematics self-efficacy (T2)	0.19^***^	0.44^***^	0.32^***^	1						
5 Mathematics self-concept (T2)	0.09^***^	0.37^***^	0.23^***^	0.58^***^	1					
6 Mathematics self-confidence (T2)	0.09^***^	0.35^***^	0.24^***^	0.59^***^	0.89^***^	1				
7 Mathematics anxiety (T2)	−0.10^***^	−0.28^***^	−0.14^***^	−0.29^***^	−0.46^***^	−0.31^***^	1			
8 SEC—responsible decision-making (T2)	0.17^***^	0.33^***^	0.27^***^	0.40^***^	0.21^***^	0.23^***^	−0.17^***^	1		
9 SEC—social awareness (T2)	0.17^***^	0.24^***^	0.23^***^	0.37^***^	0.20^***^	0.22^***^	−0.10^***^	0.50^***^	1	
10 SEC—self-management (T2)	0.16^***^	0.24^***^	0.22^***^	0.42^***^	0.23^***^	0.25^***^	−0.12^***^	0.52^***^	0.75^***^	1
11 SEC—relationship skills (T2)	0.18^***^	0.27^***^	0.22^***^	0.36^***^	0.20^***^	0.22^***^	−0.14^***^	0.47^***^	0.57^***^	0.53^***^

^***^p < 0.001. The same as below.

Mathematics self-efficacy showed moderate correlations with mathematics self-concept, mathematics self-confidence, mathematics achievement, and subdimensional SEC scores (*r* = 0.36–0.59, *p* < 0.001). A moderate negative correlation was observed between mathematics self-concept and mathematics anxiety (*r* = −0.46, *p* < 0.001), whereas mathematics self-concept was highly correlated with mathematics self-confidence (*r* = 0.89, *p* < 0.001). Positive correlations between children's perceived parental educational aspirations, educational aspirations, mathematics achievement, and subdimensional SEC scores with the four components of mathematics self-beliefs were revealed (|*r|* = 0.09–0.42, *p* < 0.001).

### 3.2 Mediation model results

SEM was conducted with children's perceived parental educational aspirations as the independent variable, mathematics self-beliefs as the mediator, and children's educational aspirations, mathematics achievement, and SEC as the dependent variables. The mediation model demonstrated good model fit (χ^2^/*df* = 3.43; RMSEA = 0.040, CFI = 0.927; TLI = 0.914; SRMR = 0.057). The model explained 21% of the variance in children's educational aspirations, 33% in mathematics achievement, and 31% in SEC.

**Figure 2:**
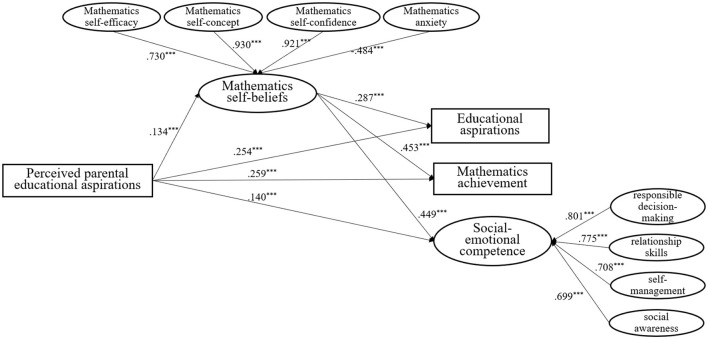
Path diagram: the mediation of mathematics self-beliefs on predicting relationships between perceived parental educational aspirations and primary school children's educational aspirations, mathematics achievement and SEC. ***p < 0.001.

The standardized regression results are shown in Figure 2 (control variables not shown for clarity). Gender differences emerged in educational aspirations (β = 0.16, *p* < 0.001), mathematics achievement (β = 0.07, *p* < 0.05) and SEC (β = 0.09, *p* < 0.01), with girls scoring higher in all three domains. Children's perceived parental educational aspirations significantly and positively predicted mathematics self-beliefs (β = 0.13, *p* < 0.001), educational aspirations (β = 0.25, *p* < 0.001), mathematics achievement (β = 0.26, *p* < 0.001), and SEC (β = 0.14, *p* < 0.001). Mathematics self-beliefs were also positively associated with educational aspirations (β = 0.29, *p* < 0.001), mathematics achievement (β = 0.45, *p* < 0.001), and SEC (β = 0.45, *p* < 0.001). The Sobel test confirmed significant mediation effects of children's mathematics self-beliefs on educational aspirations, mathematics achievement and SEC (effect = 0.07, SE = 0.02, *p* < 0.001 for educational aspirations; effect = 0.10, SE = 0.02, *p* < 0.001 for mathematics achievement; effect = 0.11, SE = 0.02, *p* < 0.001 for SEC). To further validate these pathways, the bootstrap analyses with 10,000 resamples were conducted, producing bias-corrected confidence intervals that supported the significance of all indirect effects (effect = 0.04, 95% CI [0.02, 0.06] for educational aspirations, effect = 0.06, 95% CI [0.03, 0.09] for mathematics achievement, effect = 0.06, 95% CI [0.03, 0.09] for SEC). These results indicate that children's perceived parental educational aspirations not only directly predict their educational aspirations, mathematics achievement, and SEC, but also exert indirect effects through the mediation of mathematics self-beliefs. These findings provide empirical support for all three research questions of the current study.

## 4 Discussion

The current study investigated the relationship between children's perceived parental educational aspirations and their subsequent educational aspirations, mathematics achievement, and SEC, as well as the meditation role of mathematics self-beliefs. The results showed that children's perceived parental educational aspirations directly enhanced their educational aspirations, mathematics achievement, and SEC, and also exerted indirect effects through mathematics self-beliefs. These findings provide new insights into the multifaceted benefits of children's perceived parental educational aspirations for their development.

### 4.1 Benefits of perceived parental educational aspirations

Children's perceived parental educational aspirations positively predicted their educational aspirations. This finding underscores the critical role of parents in shaping children's educational aspirations (Froiland, 2021), as well as the importance of children's perceptions of their parents' aspirations. Children's beliefs about their academic competence and the value of education are shaped by parents' views of their academic competence and educational aspirations (Fredricks and Eccles, 2002). However, it is the perception of these parental aspirations that is particularly significant, as it constitutes the foundational first step in the internalization process. Without accurate perception, there is no meaningful content for children to accept, rendering parental influence mechanistically impossible, as outlined in the two-step model of value transmission (Grusec and Goodnow, 1994). It underscores that while high parental aspirations are necessary, they are insufficient unless accurately perceived by children—perception acts as the critical mediating pathway through which intergenerational values are transmitted.

On the other hand, the results revealed statistically significant differences in children's perceived parental educational aspirations between completers (participants in both T1 and T2) and non-completers (T1 only), suggesting that children's perceptions of parental aspirations may play a role in sustaining educational engagement, albeit with a small effect size. These findings highlight the multifaceted advantages of children's perceived parental educational aspirations, which not only serve as positive predictors of their beliefs (e.g., educational aspirations), but also support their behavioral engagement over time. This provides new insights into the broader developmental benefits of children's perceived parental educational aspirations.

Moreover, parents' educational aspirations have been shown to promote children's academic progress (Lekfuangfu and Odermatt, 2022). The present study found that children's perceived educational aspirations positively predicted their mathematics achievement. This suggests that when children perceive their parents' educational aspirations, such perceptions exert a comparable facilitating role on their subsequent mathematics achievement. This finding aligns with the EVT, which posits that children's motivation to learn is shaped not only by their perceived academic competence but also by their parents' perceptions and attitudes toward education (Eccles and Wigfield, 2002; Fredricks and Eccles, 2002). By demonstrating that children's perceptions of parental educational aspirations contribute to academic success, the current study extends prior research and underscores the developmental significance of perceived parental educational aspirations.

Furthermore, the present study found that children's perceived parental educational aspirations positively predicted their SEC, highlighting that the benefits of perceived parental educational aspirations transcend academic outcomes to encompass social and emotional development. When children perceive high parental educational aspirations, this often reflects high-quality parent-child interactions—such as scaffolded learning support and goal-oriented communication—which foster a supportive family environment conducive to both cognitive abilities and SEC (e.g., Chen et al., 2023; Tang et al., 2023; Wang et al., 2022). Prior research also suggests that children exposed to positive parenting styles tend to exhibit better social adjustment, thereby promoting their social-emotional development (Lee et al., 2021; Ndengeyingoma et al., 2024). These findings enrich the literature by elucidating the broader developmental advantages of perceived parental educational aspirations and clarifying their link with SEC.

### 4.2 The mediating role of mathematics self-beliefs

The current study revealed that children's perceived parental educational aspirations enhanced their educational aspirations, mathematics achievement, and SEC through the mediation of mathematics self-beliefs. Parents serve as a primary source of support and value transmission, and when children perceive their parents' educational aspirations, this perception strengthens their positive self-evaluations, consistent with the EVT (Eccles and Wigfield, 2002; Fredricks and Eccles, 2002). This extends the documented benefits of perceiving parental aspirations by showing that such perceptions operate through mathematics self-beliefs. Positive self-evaluations, in turn, motivate children to engage more actively in learning, set higher educational goals, and develop stronger social and emotional skills. These findings underscore the multifaceted role of mathematics self-beliefs in shaping both academic and socio-emotional outcomes.

The results demonstrate that when children perceive their parents' educational aspirations as supportive and encouraging, this perception initiates a virtuous cycle in the formation of positive mathematics self-beliefs. Specifically, such perceived aspirations: (1) foster strong mathematics self-beliefs that motivate students to set ambitious learning goals and sustain engagement in academic activities, thereby enhancing scholastic performance; and (2) promote the development of SEC by supporting adaptive social interactions and effective emotional regulation. This aspiration-belief-development mechanism appears to function across both cognitive and non-cognitive domains, highlighting the pervasive influence of perceived parental educational aspirations on children's development. These findings contribute to new evidence to understanding the mechanisms through which perceived parental educational aspirations shape children's development.

### 4.3 Limitations and future directions

Several limitations should be acknowledged. First, although the two-wave longitudinal design advances beyond cross-sectional research, attrition and unmeasured confounding factors (e.g., teachers' instructional quality and expectations) may have influenced the results. Future studies should incorporate classroom-level variables and adopt multi-wave designs to better capture developmental trajectories over time. Second, the Cronbach's alpha coefficients of the subscales of SEC were relatively low, an issue that was also present in the original scale developed by Mantz et al. (2016). They considered that only three items per subscale was the important reason. Future studies could develop scales with more than three items per subscale, thereby providing a basis for the interpretation and use of subdimensional SEC scores. Third, children's perceived parental educational aspirations were measured by a single-item scale, which restricts the assessment of its psychometric properties, particularly reliability and validity. Developing a multi-item scale would allow for more rigorous validation and offer a basis for evaluating the adequacy of the single-item scale. Fourth, this study examined only children's perceived parental educational aspirations without considering potential discrepancies from parents' actual educational aspirations. Future research that investigates these differences could provide deeper insight into how parental educational aspirations—both perceived and actual—jointly shape children's development.

### 4.4 Implications

The findings of this study provide actionable implications for multiple stakeholders. For parents, it is crucial to communicate educational aspirations clearly through supportive dialogue (e.g., emphasizing effort rather than outcomes) and high-quality interactions such as scaffolded learning. These practices can strengthen children's perceptions of parental aspirations and enhance their self-beliefs. For educators, these insights can be leveraged to design parent workshops on growth-mindset communication and to incorporate classroom activities that prompt students to reflect on family educational values, thereby fostering stronger alignment between home and school environments. For policymakers, the evidence underscores the importance of funding community-based programs that reduce socioeconomic disparities in aspiration transmission, for example, by training liaisons to support low-income families in effectively articulating their educational expectations.

## 5 Conclusion

In conclusion, children's perceived parental educational aspirations positively predict their own educational aspirations, mathematics achievement, and SEC, with mathematics self-beliefs functioning as a key mediating mechanism. These findings highlight both the predictive value of perceived parental educational aspirations and the central role of self-beliefs across multiple domains of children's development. The results suggest that when parents convey high aspirations in a supportive manner and children accurately perceive these aspirations, positive self-evaluations are fostered. Such perceptions, in turn, promote children's subsequent educational aspirations, mathematics achievement, and SEC.

## Figures and Tables

**Figure A1:**
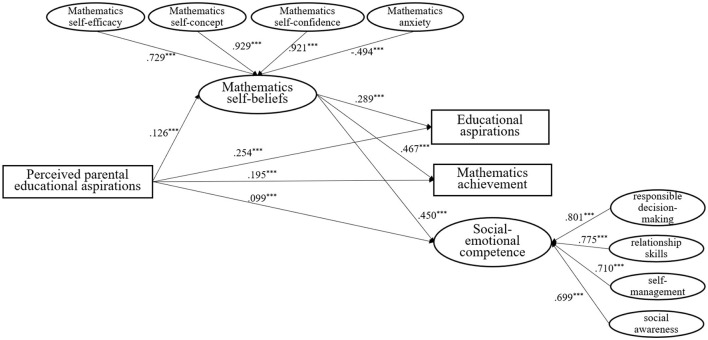
Path diagram: the mediation of mathematics self-beliefs on predicting relationships between perceived parental educational aspirations and primary school children's educational aspirations, mathematics achievement and SEC (excluding outliers). ***p < 0.001.

## Data Availability

The data analyzed in this study is subject to the following licenses/restrictions: The original data are available on reasonable request from the corresponding author. Requests to access these datasets should be directed to yehuiwang@bnu.edu.cn.

## Appendix

The mediation model (excluding outliers) demonstrated good model fit (χ^2^/*df* = 3.42; RMSEA = 0.040, CFI = 0.926; TLI = 0.914; SRMR = 0.056). The model explained 18% of the variance in children's educational aspirations, 31% in mathematics achievement, and 30% in SEC. The standardized regression results are shown in Figure A1 (control variables not shown for clarity). Gender differences emerged in educational aspirations (β = 0.17, *p* < 0.001), mathematics achievement (β = 0.10, *p* < 0.05) and SEC (β = 0.11, *p* < 0.01), with girls scoring higher in all three domains.

Children's perceived parental educational aspirations significantly and positively predicted mathematics self-beliefs (β = 0.13, *p* < 0.001), educational aspirations (β = 0.20, *p* < 0.001), mathematics achievement (β = 0.25, *p* < 0.001), and SEC (β = 0.10, *p* < 0.001). Mathematics self-beliefs were also positively associated with educational aspirations (β = 0.29, *p* < 0.001), mathematics achievement (β = 0.47, *p* < 0.001), and SEC (β = 0.45, *p* < 0.001).

The Sobel test confirmed significant mediation effects of children's mathematics self-beliefs on educational aspirations, mathematics achievement and SEC (effect = 0.04, SE = 0.01, *p* < 0.001 for educational aspirations; effect = 0.06, SE = 0.02, *p* < 0.001 for mathematics achievement; effect = 0.06, SE = 0.02, *p* < 0.001 for SEC).
